# A novel analytical method to assess the effect of imipenem/cilastatin on liver function laboratory indexes in Chinese underage inpatients: Probability distribution curve

**DOI:** 10.1371/journal.pone.0224352

**Published:** 2019-10-24

**Authors:** Le Zou, Fanqi Meng, Weici Wang, Qianqian Ye, Lin Hu, Taoming Li, Tao Yin

**Affiliations:** 1 Pharmacy Department, Xiangya Hospital, Central South University, Changsha, Hunan Province, China; 2 The Seventh Affiliated Hospital, Sun Yet-sun University, Shenzhen, Guangdong Province, China; 3 Xiangya School of Pharmaceutical Sciences, Central South University, Changsha, Hunan Province, China; 4 Pharmacy Department, Hubei University of Science and Technology, Xianning, Hubei Province, China; 5 Pharmacy Department, The Fourth Hospital of Changsha, Changsha, Hunan Province, China; Dokkyo Medical University, JAPAN

## Abstract

**Objectives:**

The primary objective of this study was to establish a novel method to assess the effect of imipenem/cilastatin (IMP) on liver function laboratory indexes in Chinese underage inpatients (inpatients aged <18 year-old).

**Methods:**

A retrospective study was conducted in 188 underage inpatients who received IMP in Xiangya Hospital from January 2016 to April 2018. Demographic data and clinical information of these inpatients were collected. As there was no reference interval of minors, the occurrence of abnormal liver function was estimated by that of adults, temporarily. A new concept (mean-variance induced by drug, MVID) was introduced to analyze the characteristics of total bilirubin (TBil), direct bilirubin (DBil), alanine aminotransferase (ALT) and aspartate aminotransferase (AST). Effect of MVID of TBil, DBil, ALT and AST in different patients (aged<1 year old and aged ≥ 1 year old) were compared by Mann-Whitney U test.

**Results:**

Estimating by reference intervals of adults, 57.4% underage inpatients (108/188) had abnormal liver function. According to the probability distribution curve of MVID, IMP can cause the increase of AST in 24% (0.62–0.38) Chinese underage inpatients, and the increase of ALT in 20% (0.60–0.40) Chinese underage inpatients. And liver protecting drugs can decrease MVID of ALT and AST. There were not statistically significant differences in MVID of TBil, DBil, ALT and AST in different patients (aged<1 year old and aged ≥ 1 year old); *P* value was 0.711, 0.734, 0.067 and 0.086, respectively.

**Conclusion:**

IMP can affect the liver function of 20–24% Chinese underage inpatients mainly by increasing the AST and ALT. IMP may induce hepatocellular injury, but not cholestasis. And liver protecting drugs can reverse the side effects caused by IMP. Age may not affect the effect of IMP on liver function.

## Introduction

Imipenem is the leading compound of the carbapenems and has broad antibacterial spectrum and highly potent therapeutic effects [1–4]. Now, imipenem is widely used in large comprehensive hospitals in China [5, 6]. It is often used in combination with cilastatin, a renal dehydropeptidase inhibitor, to prevent its rapid metabolism in kidneys and reduce the renal toxicity of its metabolites [7].

Though the liver safety of imipenem/cilastatin (IMP) is generally believed to be reliable and it may only cause mild liver transaminase elevation [8, 9], the hepatotoxicity of IMP has been reported multiple times [10–13]. It has been also found that IMP could easily lead to abnormal liver function and even serious liver function damage in Chinese underage inpatients (inpatients aged <18 year-old) [14]. Due to the limited blood sampling, the ethical hurdle for patient enrollment in clinical studies, and potential adverse effects on growth, it is difficult to evaluating the hepatotoxicity of IMP in underage inpatients. Meanwhile, there was no reference interval for common clinical biochemistry tests for Chinese underage inpatients yet [15]. Chinese clinicians are often stumped when the liver transaminase abnormalities appear in underage patients after administration of IMP, which can greatly affect clinical medication decision and patient’s compliance. Thus, more feasible approach is needed to assess hepatotoxicity of IMP in underage inpatients.

Nowadays, a large number of historical cases and data have been accumulated in hospitals, which have great scientific research value and could be used for solving the above problem. However, there are too many random noises in clinical data to analyze and the standards of each case are not unified. It is difficult to analyze these massive disordered clinical data using traditional retrospective study. Noise reduction is a basic problem of machine learning, whose principle is to get the target model from the original data so as to separate noise from the data [16]. It may be a new exploration direction to further analyze clinical data.

In our study, 1361 Chinese underage inpatients admitted by Xiangya Hospital of Central South University, who received IMP from January 2016 to April 2018 were screened. Based on the reference intervals of adults, the condition of abnormal liver function in these Chinese underage inpatients was analyzed. Then a novel clinical data analysis method (probability distribution curve) was established and the relationship between the abnormal liver function and IMP was studied. Our study provides reasonable recommendations to clinicians. And the method we established is also a novel exploration to analyze hospital historical data.

## Subjects and methods

### 2.1 Subjects

This study was carried out in Chinese underage inpatients admitted by Xiangya Hospital of Central South University from January 2016 to April 2018. Inclusive criteria: (1) The patient’s age is less than 18 year-old. (2) There was a clear indication of the medication of IMP. The duration of medication was not less than 3 days. (3) Medication records were intact. (4) At least 2 liver function laboratory tests were performed during the hospitalization. Exclusive criteria: (1) There was a history of liver disease (viral hepatitis, hyperbilirubinemia, cirrhosis, liver trauma, etc.) prior to the medication of IMP. (2) Patients treated with hepatotoxic drugs or chemotherapy. (3) Patients with diagnosis of multiple organ dysfunction syndrome. (4) Patients with severe abnormalities (alanine aminotransferase, ALT >100U/L or aspartate aminotransferase, AST >100U/L) before the treatment with IMP. (5) Patients treated with liver protecting drugs at least one week before or during administration of IMP.

Through the hospital information system, basic information of patients who were eventually included was recorded by two researchers. The information included date of birth, sex, weight (in kilograms), the starting and ending dates of treatment with IMP, method of administration, dosage (in grams), and frequency of administration (several times a day). Besides, the data and results of liver function test, including liver enzymes and/or bilirubin in plasma, during the hospitalization were also recorded.

### 2.2 Definition of abnormal liver function

As there was no laboratory reference interval for underage patients in China [15], the adults’ reference intervals for common clinical biochemistry tests were used for reference to define the liver function of underage inpatients temporarily. The reference intervals in Xiangya Hospital, which are based on the reference intervals of adults [15], are ALT, male (9–50 U/L), female (7–40 U/L); AST, male (15–40 U/L), female (13–35 U/L); total bilirubin (TBil), 1.7–17.1 μmol/L; direct bilirubin (DBil), 0–6.8 μmol/L; alkaline phosphatase (ALP), 45–125 U/L.

Definition of abnormal liver function: at least one of ALT, AST, ALP, TBil and DBil was above the upper limit (ULN) of the reference value. Abnormal liver function was graded by classification criteria in NCI.CTC v4.03 [17]. The determination of abnormal liver function level of every patient was subject to the highest one in 4 indexes.

The Naranjo adverse reaction scale [18] was used to grade the included patients, and the possibility of variances of liver function laboratory indexes induced by IMP was evaluated according to the score. The possibility was assigned to four categories from the total score as follows: Definite, ≥9; Probable, 5 to 8; Possible, 1 to 4; Doubtful, ≤0.

### 2.3 Definition and calculation of MVID

As described above, the clinical data was influenced by too many factors to analyze. In order to quantify the effect of IMP on liver function (bilirubin and transaminase), the commonalities in data (use of IMP and patients’ age <18 year-old) were extracted as describe in “**2.1 Subjects**”. After selection, the inpatients included were all only administrated with IMP, and didn’t have abnormal liver function before administration. Considering that the side effects of the IMP may be dose-related [19, 20] and the clinical dosage of IMP is usually related with patient’s weight, daily drug dosage (Single dose * Medication frequency / Weight) was used to analyze the side effects of IMP.

However, there were still many other factors that interfere with liver function. Parts of them were common factors, such as weight, administration dosage, interval of drug administration, duration of medication. For excluding the influences of common factors, a new concept was introduced, which was the mean-variance induced by drug (MVID). MVID was used to represent the influence of the unit daily dosage of drug (one gram and once a day) on the laboratory characteristics of liver function of the unit weight patients (one kilogram) in one day, which can be calculated by formula (1):

$$MVID=\frac{Index\;variance\;of\;two\;liver\;function\;tests\times Weight\;}{Single\;dose\times Medication\;frequency\times The\;medication\;time\;during\;the\;test\;interval}$$ (1)

The index variances of liver function tests were about TBil, DBil, ALT and AST. The MVID of TBil, DBil, ALT and AST of each patient under the same dosing regimen were calculated respectively. The unit of MVID is U*kg/ (L*g*day) or μmol*kg/ (L*g*day). For the sake of simplicity, the unit will not be added when referring to MVID.

For patients whose liver function had been examined within 3 days prior to IMP administration, the variation of liver function laboratory indexes before and after IMP administration were used to calculate the MVID. For patients whose liver function was unknown within 3 days prior to IMP administration, the variation of liver function laboratory indexes between two laboratory examinations during IMP administration were used to calculate the MVID.

### 2.4 Statistical analysis

Normal distribution data were presented as means ±standard deviation (SD). While, non-normal distribution data were described by median and interquartile range (IQR). The scatter plots were drawn by ‘Graphpad Prism 7.0’. The probability distribution curve was used to study the characteristics of the data as mentioned in previous studies [21, 22]. The Seaborn module in Python was used to calculate and describe the probability distribution of data. MVID of liver function laboratory indexes in different patients (aged<1 year old and aged ≥ 1 year old) were compared using a Mann-Whitney U test. A two-sided *P* value of 0.05 was considered statistically significant.

### 2.5 Ethics

All procedures performed in studies involving human participants were reviewed by the Ethics Committee of Xiangya Hospital Central South University and were finally approved (Ethical approval documents: NO. 2018101086).

## Result

### 3.1 General condition of the 188 patients

**Fig 1:**
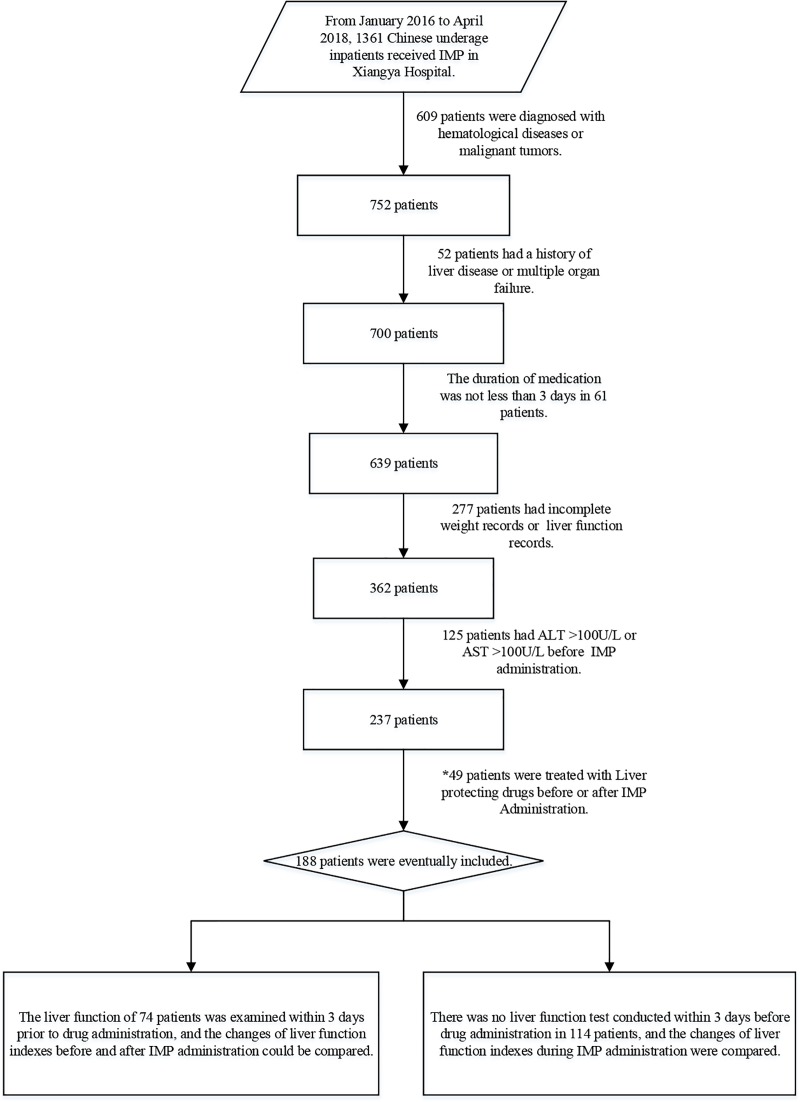
The screening process for eligible patients. 188 Chinese underage inpatients were included from 1361 patients, who received administration of IMP from January 2016 to April 2018. * 49 patients, who were treated with liver protecting drugs, were not included into the 188 patients, but were analyzed separately to investigate the effect of liver protecting drugs on MVID of AST and ALT.

From January 2016 to April 2018, 1361 Chinese underage inpatients received IMP, and 188 patients were included in this study as required. The screening process for patients was shown in Fig 1. Besides, the general conditions of 188 patients were shown in Table 1.

**Table 1:** Characteristics of the included 188 Chinese underage inpatients.

Characteristic	Case (Constituent ratio, %)
Demographic	
Male	119 (63.3)
age <1 year old	110 (58.5)
1 year old ≤ age <3-year old	14 (7.4)
3-year old ≤age <6-year old	12 (6.4)
6-year old ≤age <12-year old	29 (15.4)
12-year old ≤age <18-year old	23 (12.3)
Clinical	
Infection type ^a^	
Pulmonary infection	67 (35.6)
Septicemia	63 (33.5)
Sepsis	29 (15.4)
Intracranial infection	9 (4.8)
Skin and soft tissue infection	11 (5.9)
Enteritis	6 (3.2)
Others ^b^	8 (4.3)
Score of Naranjo adverse reaction scale	
5–8	177 (94.1)
≥9	11 (5.9)

a: Due to the poor physiological status of some patients and the existence of multiple infections, the cumulative percentage is more than 100%.

b: Others were pancreatitis, perianal infection, renal abscess, abdominal infection and cystitis. The number of such patients was few, so they were analyzed together.

The variation of liver function laboratory indexes in 188 Chinese underage inpatients was shown in Table 2.

**Table 2:** Variation of liver function laboratory indexes in 188 Chinese underage inpatients.

Liver function laboratory indexes	For 74 patients whose liver function had been examined within 3 days prior to IMP administration		For 114 patients whose liver function had not been examined within 3 days prior to IMP administration	
	Before IMP administration Mean (SD)	After IMP administration Mean (SD)	First liver function test after IMP administration Mean (SD)	Follow-up liver function during IMP administration* Mean (SD)
ALT (U/L)				
Male	14.7 (16.3)	15.4 (19.8)	19.1 (15.2)	17.1 (10.6)
Female	15.5 (16.2)	28.2 (68.4)	10.6 (6.1)	15.2 (6.7)
AST (U/L)				
Male	41.8 (22.2)	43.2 (42.8)	35.4 (17.6)	36.2 (17.9)
Female	38.8 (44.7)	62.5 (26.6)	34.3 (19.2)	42.5 (27.2)
ALP (U/L)	542.6 (255.8)	621.2 (333.1)	303 (209)	492.1 (328.8)
TBil (μmol/L)	37.7 (46.4)	34.6 (43.4)	53.8 (61.4)	45.5 (51.7)
DBil (μmol/L)	7.1 (8.12)	7.4 (9.1)	8.3 (7.4)	9.6 (8.9)

* The highest liver function laboratory indexes during the IMP administration should be chosen.

### 3.2 Abnormal liver function in 108 patients

108 of 188 patients had abnormal liver function after IMP administration. Table 3 showed the classification of abnormal liver function and the general situation of the 108 patients. The physiological status of 108 patients with abnormal liver function was poor, and the common complications were pulmonary infection (45.4%), septicemia (32.4%) and sepsis (13.0%). None drug withdrawal was due to abnormal liver function.

**Table 3:** Abnormal liver function and general conditions in 108 patients.

Characteristic	Cases (Constituent ratio, %)
Abnormal liver function	
Occurrence	108 (57.4)
Grading	
Grade 1	55 (50.9)
Grade 2	16 (14.8)
Grade 3	33 (30.6)
Grade 4	4 (3.7)
General conditions	
Male	71 (65.7)
age <1 year old	62 (57.4)
1 year old≤ age <3-year old	9 (8.3)
3-year old≤age <6-year old	6(5.6)
6-year old≤age <12-year old	18(16.7)
12-year old≤age <18-year old	13(12.0)
Infection type ^a^	
Pulmonary infection	49(45.4)
Septicemia	35(32.4)
Sepsis	14(13.0)
Intracranial infection	3(2.8)
Skin and soft tissue infection	5(4.6)
Enteritis	2(1.9)
Others ^b^	2(1.9)

a: Due to the poor physiological status of some patients and the existence of multiple infections, the cumulative percentage is more than 100%.

b: Others were pancreatitis, perianal infection, renal abscess, abdominal infection and cystitis. The cases of such patients were small, so they were analyzed together.

### 3.3 Probability distribution of MVID

**Fig 2:**
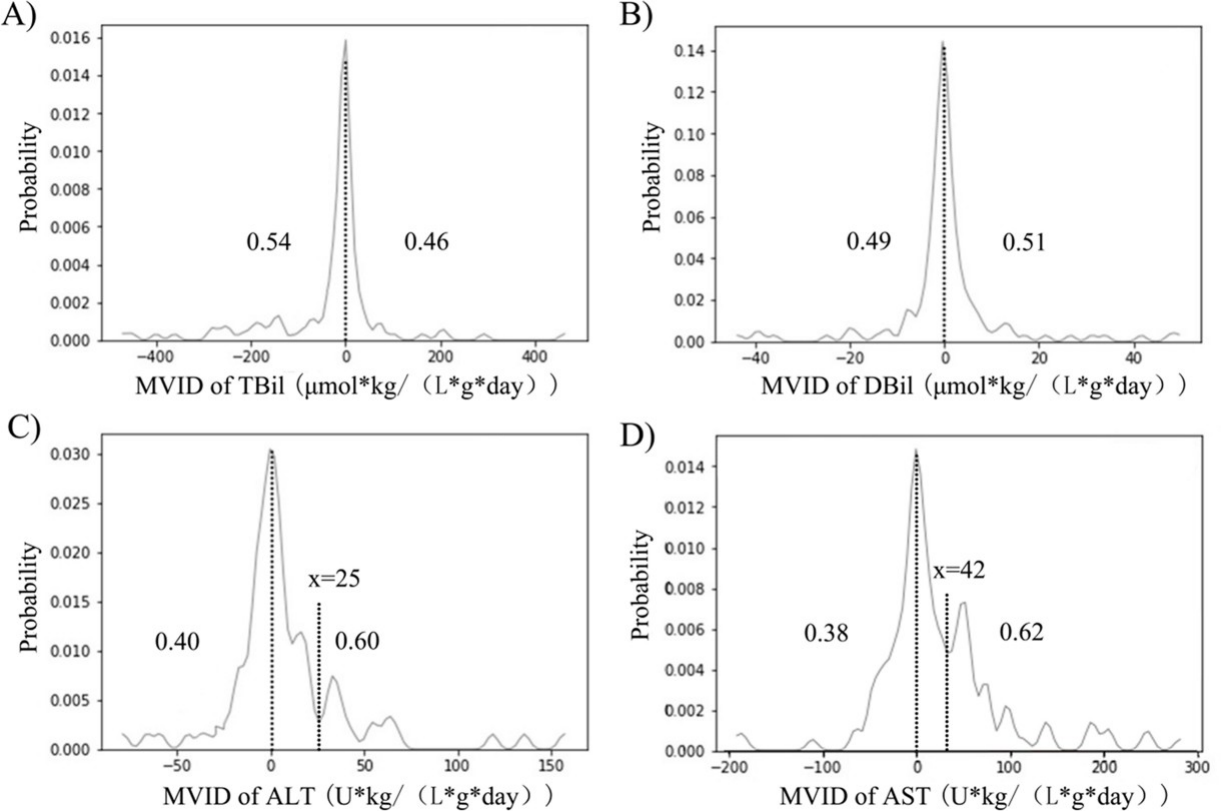
**Probability distribution of MVID of liver function laboratory indexes (A. TBil; B. DBil; C. ALT; D. AST).** The horizontal coordinate represented the value of MVID. The y-coordinate represented the probability of a certain MVID value. The area of the under curve (AUC) of the left side and right side were shown in each picture.

As was shown in Fig 2, the probability distribution of the MVID of DBil and TBil was a symmetric distribution with a mean value of 0. However, in the probability distribution of AST and ALT, the curve was not symmetric around ‘x = 0’, but a positive deviation distribution. In Fig 2D, the area under the curve (AUC) of both sides was calculated. In the probability distribution curve of AST, the left AUC was 0.38, and the right was 0.62. On the right of ‘X = 42’, its probability distribution curve showed a significant increase trend, while between ‘X = 0’ and ‘X = 42’, it is still symmetrical to the left. In the probability distribution curve of ALT, the left AUC was 0.40, and the right was 0.60. And on the right of ‘X = 25’, its probability distribution curve showed a significant increase trend.

### 3.4 Effect of liver protecting drugs

As mentioned in Fig 1, 49 patients who were treated with liver protecting drugs before or after administration of IMP, were screened. Among them, 33 patients with incomplete information and inability to calculate MVID were excluded. Finally, 16 patients were included in the following analysis.

10 patients was administrated liver protecting drugs to prevent abnormal liver function. The other 6 patients were treated with liver protecting drugs for abnormal liver function. The following drugs were used: reduced glutathione (11 cases, 68.75%), compound glycyrrhizin (4 cases, 25%) and magnesium isoglycyrrhizinate (5 cases, 31.25%). Because 4 patients were treated with two liver protecting drugs, the cumulative percentage of the drugs was more than 100%.

**Fig 3:**
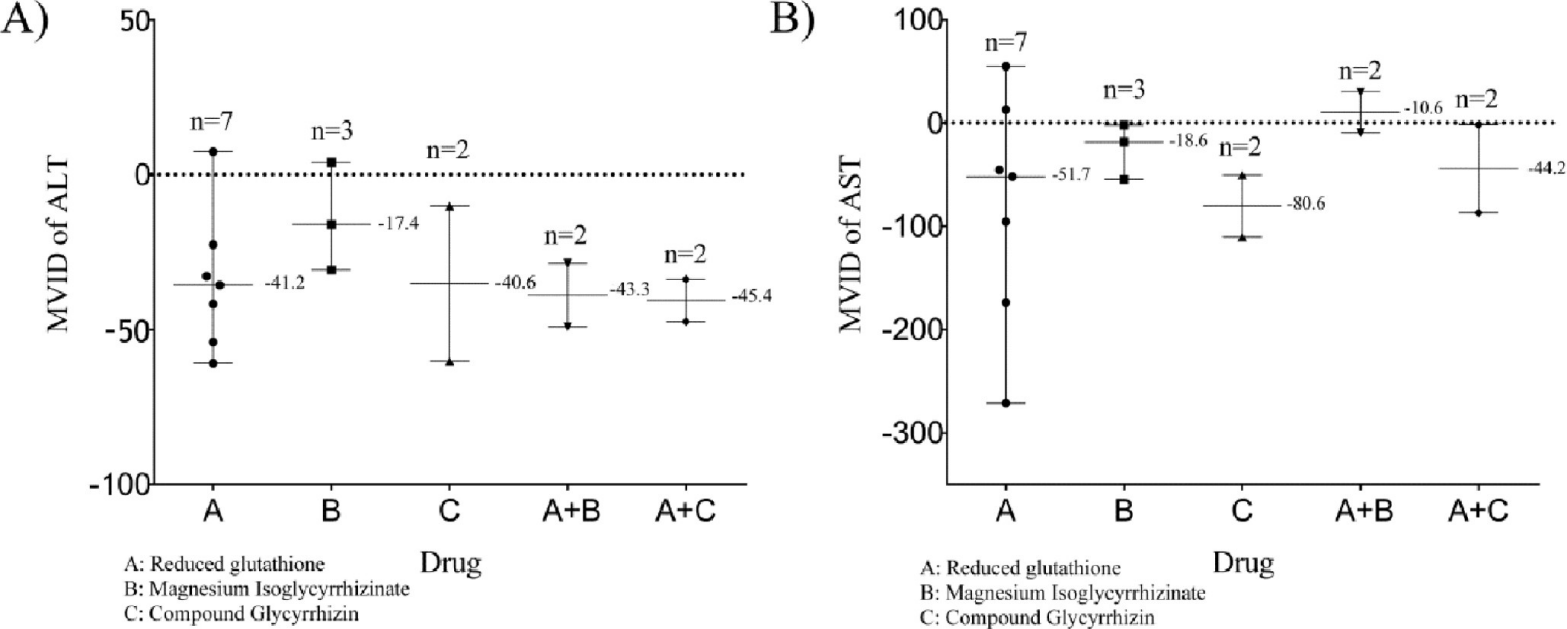
**The distribution of MVID after intervention of liver protecting drugs (A. ALT; B. AST).** The “y = 0” was marked by a dotted line. From top to bottom, the three solid horizontal lines respectively represent the maximum MVID, the median MVID and the minimum MVID. The number of patients and the median value of MVID were respectively marked above the top line or the middle line. A+B and A+C represented combination therapy.

Fig 3 showed MVID of ALT and AST in 16 patients treated with liver protecting drugs. It can be seen that the MVID of AST and ALT were generally low in patients with protecting liver drug. In addition to 3 patients who were treated with liver protecting drugs to improve liver function, the MVID of AST was less than 0 in the remaining 13 patients. 14 patients of the MVID of ALT was less than 0 after administration. The remaining 2 patients were also significantly reduced.

After using liver protecting drugs, the MVID of AST in 81.3% (13/16) underage patients and the MVID of ALT in 87.5% (14/16) underage patients changed from positive value to negative value, respectively.

### 3.5 Influence of age on occurrence of IMP side effect

Most patients (58.5%) in this study were under 1 year old. To investigate whether age affects the side effect of IMP on liver function, MVID of TBil, DBil, ALT and AST in different patients (aged<1 year old and aged ≥ 1 year old) were compared by Mann-Whitney U test. But there were not statistically significant differences; *P* value was 0.711, 0.734, 0.067 and 0.086, respectively.

## Discussion

### 4.1 General condition of 188 enrolled patients

As shown in Table 1, the median age of 188 patients was 324 days (16 days, 2501 days). The median weight was 4.57kg (1.48kg, 20.00kg), and the median score of Naranjo adverse reaction scale was 6 (5, 8). The types of infections in patients are commonly pulmonary infections (67, 35.6%), septicemia (63, 33.5%) and sepsis (29, 15.4%), rare intracranial infections (9, 4.8%), skin and soft tissue infections (11, 5.9%), enteritis (6, 3.2%), and other infections (8, 4.3%).

It was also found that ALT, AST and ALP were increased in different degrees after IMP administration according to Table 2. TBil and DBil showed little change before and after IMP administration. The baseline ALP of patients was high. This may be because most of patients in this study were younger than 1 year old, whose bones develop rapidly, resulting in high ALP. However, only 27 of 188 patients underwent at least two ALP tests before and after IMP administration. Since there were few valid data on ALP, its MVID was not analyzed in this study.

### 4.2 Occurrence of abnormal liver function

Due to the lack of reference interval for laboratory indexes for Chinese minors [15], reference intervals for adults was used to assess the abnormal liver function of minors temporarily. 188 patients were evaluated according to the score of Naranjo adverse reaction scale. It was found that variances of liver function laboratory indexes of 11 patients were probably induced by IMP (the score of Naranjo adverse reaction scale was 5 to 8). And variances of liver function laboratory indexes of the other 177 patients were definitely induced by IMP (the score of Naranjo adverse reaction scale was ≥9).

57.4% patients (108/188) appeared abnormal liver function after IMP administration. This is much higher than the rate of abnormal liver function described in the drug specification [9] and in previous research. The rate of abnormal liver function caused by IMP was reported as 1.7%, 1/56 [23]; 8.5%, 11/30 [24]; 28.6%, 6/21 [25].

Even abnormal liver function of 37 patients in 108 patients (34.3%) developed Grade 3 or Grade 4. The physiological status of 108 patients with abnormal liver function was poor, and the common complications were pulmonary infection (45.4%), septicemia (32.4%) and sepsis (13.0%). None drug withdrawal was due to abnormal liver function.

The occurrence rate of abnormal liver function in patients with poor physiological status in our study might be overestimated, because 50.9% patients was considered Grade one according to NCI.CTC v4.03, which indicates these patients were with a slight increase in AST or ALT. In order to reduce errors caused by different criteria between our study and previous studies [23–25], the new concept MVID was introduced to further analyze the side effect of IMP on liver function of patients.

### 4.3 Probability distribution of MVID

Because the clinical data was too messy to analyze, it was preprocessed in two steps. The standard of data was unified in “**2.1 Subjects**”, 1361 clinical cases were screened out and the patients whose abnormal liver function was only caused by IMP were selected. The commonalities of the data were extracted. Then MVID was calculated and used to exclude the effect of common factors on liver function, as described above. However, there were still many confounding factors that had not been filtered, such as physical and psychological condition, genetic background, living habit, diet, other possible hepatotoxic drugs and so on. For these confounding factors, the probability distribution curve of data was used to analyze data characteristics. The principle is that when the amount of data is large, the unique commonality in the data will influence the probability distribution curve of the data directionally. Meanwhile, the random noise caused by confounding factors can only affect the heterogeneity of the data distribution.

In Fig 2, the center of symmetric distribution describes the average of the effects, while its width describes the degree of heterogeneity. As IMP has no liver-protection effect, the variables studied are impossible to have a negative deviation distribution. The part of curve on the left of ‘X = 0’ was caused by non-directional confounding factors and normal fluctuations, which also exist on the right of ‘X = 0’. It means that after medication of IMP, the common character of data (influence of IMP) can affect almost 20% (0.60–0.40, Fig 2C) Chinese underage inpatients’ liver function mainly by increasing the ALT, and 24% (0.62–0.38 Fig 2D) Chinese underage inpatients’ liver function mainly by increasing the AST which also has been reported by Sun T. and his colleagues [26].

Transaminase has been widely used in clinical evaluation of liver diseases as a main serological marker [27–29]. Abnormal elevation of transaminase often suggests liver injury [30]. Considering the particularity of underage inpatients, the side effect of IMP in inpatients should be paid more attention in clinical practice.

### 4.4 Possible mechanism of effects of IMP on liver function

According to results of probability distribution of MVID of liver function laboratory indexes, IMP mainly led to abnormal liver function by increasing transaminases levels. It’s suggested that IMP may induce hepatocellular injury, but not cholestasis. Due to the lack of specific mechanism studies on the impact of IMP on liver function, our study only reasonably speculates the possible mechanism: It has been reported that the clearance rate of cilastatin greatly reduced after the application of IMP in patients with hepatic insufficiency [31]. This indicates that part of cilastatin is still metabolized and excreted by the liver. Thus, the increase of transaminases in Chinese underage inpatients caused by IMP may be related to the metabolism of the liver and excretion of cilastatin. In addition, it has been found that carbapenems can activate the glutaraldehyde acidification reaction of sodium valproate in liver, thereby reducing the level of sodium valproate in plasma [32]. This also implies that IMP is likely to affect the content of transaminases in the body by affecting the glutaraldehyde acidification reaction in liver. But, we can’t exclude the possibility that IMP can influence the body's immune response, reduce the body's antioxidant ability, then resulting in mitochondrial oxidative damage and leading to damage of mitochondria.

However, in order to reduce the risk of abnormal liver function in patients after the application of IMP and to provide a strong experimental basis, systematic basic experiments are urgently needed to verify the above conjectures in order to make up for the gaps in the current field. The reference interval for common clinical biochemistry tests for Chinese underage inpatients also should be supplemented.

### 4.5 Effect of liver protecting drugs

Whether using liver protecting drugs in inpatients with IMP was a widespread confusion among clinicians, which may have an impact on clinical decision and patients’ compliance. According to result of Fig 3, liver protecting drugs can significantly reduce the MVID of AST and ALT in minor patients. After using liver protecting drugs, the MVID of AST in 81.3% (13/16) underage patients and the MVID of ALT in 87.5% (14/16) underage patients changed from positive value to negative value, respectively. It was proved that liver protecting drugs can inhibit and even reversed the increase trend of AST and ALT.

Therapeutic application of liver protecting drugs had a significant effect. Whether to apply as prophylaxis or not depends on the patient's physical condition and economic conditions.

### 4.6 Effect of age on occurrence of IMP side effect

MVID of TBil, DBil, ALT and AST in different patients (aged<1 year old and aged ≥ 1 year old) were compared by Mann-Whitney U test. *P* value was 0.711, 0.734, 0.067 and 0.086, respectively. It’s suggested that age may not be influencing factor of effect of IMP on liver function. This may be related to the small sample size of our study. Therefore, large sample studies are needed to verify this issue.

### 4.7 Advantage of analysis of probability distribution

When studying the pharmacological effects and side effects of drugs, clinical trials are designed and conducted to observe the effects of the drug on clinical outcomes. The related variables are rigorously controlled. However, it is difficult to including special populations such as children and pregnant women in clinical trials. In the real world, the applicability of clinical trial results of general population to the special population remains to be verified. Therefore, the safety of drugs for special populations still needs to be studied and analyzed in daily clinical practice.

In a retrospective study of clinical data, principle of control is regularly used to determine whether the exposure factor has a statistically significant effect on clinical outcomes. However, in the clinical medication data, the heterogeneity of basic status and dosing regimen of each patient (the poor basic status of some patients and the existence of multiple infections) will lead to inconsistent data standards and huge data noise. The accuracy of the analysis results of traditional statistical methods will be seriously interfered (False negative results are easily produced when the data noise is large). As a result, it is difficult to make accurate judgments. When abnormal liver function or even severe liver damage (Grade 3 or Grade 4 liver function) occur in the severely infected underage patients after administration of IMP, clinicians still could not tell whether the abnormal liver function was caused by drug or other factors in the patient's own basic status.

After comparison, it can be found that if only a simple descriptive analysis was performed on patients with abnormal liver function after the use of IMP, proportion of patients with abnormal liver function was relatively high under the interference of other confounders. The prevalence rate of abnormal liver function in patients administrated with IMP in different studies was also significantly different [14, 22–24]. It may be due to inconsistencies in data recording standards and physiological status of patients in different studies, or differences in judgment criteria of abnormal liver function. Therefore, it is difficult to distinguish the occurrence of liver dysfunction due to the use of IMP or other confounders. To solve this problem, the actual effect of IMP on patients' liver function was studied from the characteristic of data distribution. Through this method, the prevalence rate of abnormal liver function is calculated as 20–24%, close to Zajac’s research results [25] but much lower than that (108/188, 57%) obtained from traditional statistical methods (mentioned in “**3.2 Analysis of abnormal liver function**”). This may be because confounding factors (the basic status of the patient, the infection itself, other associated diseases or co-administration of other drugs, etc.) have a greater impact on the results of traditional methods.

In the analysis of clinical data with non-uniform standards and large noise, except the traditional statistical significance difference analysis method, the distribution law of the data itself also can be used to make a reasonable inference of the clinical facts. Our study provide the following recommendations to clinicians: when abnormal liver function or even severe liver damage occur in the severely infected underage patients after administration of IMP, if the patient's AST and ALT significantly increased, even much higher than the upper normal limit, alternative medicines can be selected, or hepatic protection drugs can be used; if other indicators (DBil or TBil) increased, it is also necessary to comprehensively consider whether there are other reasons (the basic status of the patient, the infection itself, other associated diseases or co-administration of other drugs, etc.) of the abnormality of these laboratory indexes.

In conclusion, the incidence of abnormal liver function after IMP administration may be overestimated when only criteria of NCI.CTC v4.03 [17] were used. Analysis of MVID can be regarded as a good supplement to traditional statistical methods, and also can provide a new direction for clinical data analysis.

## Limitations

This study was a retrospective prevalence study to analyze the prevalence rate of abnormal liver function in Chinese underage inpatients with IMP at a specific time. The statistical association between exposure (IMP) and disease (abnormal liver function) can only be used as a clue and hypothesis.

Whether above effect (IMP may increase the AST and ALT of Chinese underage inpatients) is related to the dose of IMP cannot be determined by the new concept “MVID”. The sample size of this study was limited, and the conclusions of this study need to be further verified by a multi-center, larger sample controlled study.

However, our findings were not only aim to attract public attention to the liver safety of IMP in underage inpatients, but also provide a novel analytical method to analyze complex clinical data.

## Conclusion

Of 188 patients, 108 patients (57.4%) appeared abnormal liver function after IMP administration. IMP can affect the liver function of part of Chinese underage inpatients mainly by increasing the AST and ALT. And the prevalence rate of abnormal liver function caused by IMP may be considered as 20–24%, actually. IMP may induce hepatocellular injury, but not cholestasis. Liver protecting drugs can reverse the side effects caused by IMP. Age may not affect the effect of IMP on liver function.

## Supporting information

S1 Data Original data. Data related to this study can be found in S1 Data.
(XLSX)

S1 File STROBE statement. Checklist of items that should be included in reports of observational studies can be found in S1 File.
(DOCX)
