# Supplementary material for: A novel analytical method to assess the effect of imipenem/cilastatin on liver function laboratory indexes in Chinese underage inpatients: Probability distribution curve
Source: PLoS One. 2019 Oct 24;14(10):e0224352. doi: 10.1371/journal.pone.0224352 (PMC6812810; doi:10.1371/journal.pone.0224352)
Supplement: S1 File — Checklist of items that should be included in reports of observational studies can be found in S1 File. (DOCX) [file pone.0224352.s002.docx]

STROBE Statement—checklist of items that should be included in reports of observational studies

|  | Item No. | Recommendation | Page  No. | Relevant text from manuscript |
| --- | --- | --- | --- | --- |
| **Title and abstract** | 1 | (*a*) Indicate the study’s design with a commonly used term in the title or the abstract | 2 | A retrospective study was conducted in 188 underage inpatients who received IMP in Xiangya Hospital from January 2016 to April 2018. |
|  |  | (*b*) Provide in the abstract an informative and balanced summary of what was done and what was found | 2 | Demographic data and clinical information of these inpatients were collected. As there was no reference interval of minors, the occurrence of abnormal liver function was estimated by that of adults, temporarily. A new concept (mean-variance induced by drug, MVID) was introduced to analyze the characteristics of total bilirubin (TBil), direct bilirubin (DBil), alanine aminotransferase (ALT) and aspartate aminotransferase (AST). Effect of MVID of TBil, DBil, ALT and AST in different patients (aged＜1 year old and aged ≥ 1 year old) were compared by Mann-Whitney U test.  Estimating by reference intervals of adults, 57.4% underage inpatients (108/188) had abnormal liver function. According to the probability distribution curve of MVID, IMP can cause the increase of AST in 24% (0.62-0.38) Chinese underage inpatients, and the increase of ALT in 20% (0.60-0.40) Chinese underage inpatients. And liver protecting drugs can decrease MVID of ALT and AST. There were not statistically significant differences in MVID of TBil, DBil, ALT and AST in different patients (aged＜1 year old and aged ≥ 1 year old); *P* value was 0.711, 0.734, 0.067 and 0.086, respectively. |
| Introduction | | | |  |
| Background/rationale | 2 | Explain the scientific background and rationale for the investigation being reported | 2-4 | See the” Introduction” of the manuscript for detail. |
| Objectives | 3 | State specific objectives, including any prespecified hypotheses | 3 | Thus, more feasible approach is needed to assess hepatotoxicity of IMP in underage inpatients. |
| Methods | | | |  |
| Study design | 4 | Present key elements of study design early in the paper | 4-5 | See the inclusive criteria and exclusive criteria in “Subjects” for detail. |
| Setting | 5 | Describe the setting, locations, and relevant dates, including periods of recruitment, exposure, follow-up, and data collection | 4-5 | Line 84-99 |
| Participants | 6 | (*a*) *Cohort study*—Give the eligibility criteria, and the sources and methods of selection of participants. Describe methods of follow-up  *Case-control study*—Give the eligibility criteria, and the sources and methods of case ascertainment and control selection. Give the rationale for the choice of cases and controls  *Cross-sectional study*—Give the eligibility criteria, and the sources and methods of selection of participants | 4-5 | See “Subjects” for detail. |
|  |  | (*b*) *Cohort study*—For matched studies, give matching criteria and number of exposed and unexposed  *Case-control study*—For matched studies, give matching criteria and the number of controls per case | N/A | This is a cross-sectional study. |
| Variables | 7 | Clearly define all outcomes, exposures, predictors, potential confounders, and effect modifiers. Give diagnostic criteria, if applicable | 5-7 | See “**2.2 Analysis of abnormal liver function**” to “**2.3 Definition of MVID**” |
| Data sources/ measurement | 8* | For each variable of interest, give sources of data and details of methods of assessment (measurement). Describe comparability of assessment methods if there is more than one group | 4-5 | See “**2.2 Definition of abnormal liver function**” to “**2.3 Definition and calculation of MVID**” |
| Bias | 9 | Describe any efforts to address potential sources of bias | 5 | Through the hospital information system, basic information of patients who were eventually included was recorded by two researchers. |
| Study size | 10 | Explain how the study size was arrived at | 8 | From January 2016 to April 2018, 1361 Chinese underage inpatients received IMP, and 188 patients were included in this study as required. The screening process for patients was shown in Fig 1. Besides, the general conditions of 188 patients were shown in Table 1. |

Continued on next page

| Quantitative variables | 11 | Explain how quantitative variables were handled in the analyses. If applicable, describe which groupings were chosen and why | 4-6 | The reference intervals in Xiangya Hospital, which are based on the reference intervals of adults [15], are ALT, male (9-50 U/L), female (7-40 U/L); AST, male (15-40 U/L), female (13-35 U/L); total bilirubin (TBil), 1.7-17.1 μmol/L; direct bilirubin (DBil), 0-6.8 μmol/L; alkaline phosphatase (ALP), 45-125 U/L.  Definition of abnormal liver function: at least one of ALT, AST, ALP, TBil and DBil was above the upper limit (ULN) of the reference value. Abnormal liver function was graded by classification criteria in NCI.CTC v4.03 [17]. The determination of abnormal liver function level of every patient was subject to the highest one in 4 indexes.  The Naranjo adverse reaction scale [18] was used to grade the included patients, and the possibility of variances of liver function laboratory indexes induced by IMP was evaluated according to the score. The possibility was assigned to four categories from the total score as follows: Definite, ≥9; Probable, 5 to 8; Possible, 1 to 4; Doubtful, ≤0. |
| --- | --- | --- | --- | --- |
| Statistical methods | 12 | (*a*) Describe all statistical methods, including those used to control for confounding | 7 | Line 143-149 |
|  |  | (*b*) Describe any methods used to examine subgroups and interactions | 15-16 | Line 251-261 |
|  |  | (*c*) Explain how missing data were addressed | 8 | From January 2016 to April 2018, 1361 Chinese underage inpatients received IMP, and 188 patients were included in this study as required. The screening process for patients was shown in Fig 1. Besides, the general conditions of 188 patients were shown in Table 1. |
|  |  | (*d*) *Cohort study*—If applicable, explain how loss to follow-up was addressed  *Case-control study*—If applicable, explain how matching of cases and controls was addressed  *Cross-sectional study*—If applicable, describe analytical methods taking account of sampling strategy | N/A | We screened underage inpatients at specific time periods according to inclusion and exclusion criteria, without sampling. |
|  |  | (*e*) Describe any sensitivity analyses | 16 | For these confounding factors, the probability distribution curve of data was used to analyze data characteristics. The principle is that when the amount of data is large, the unique commonality in the data will influence the probability distribution curve of the data directionally. Meanwhile, the random noise caused by confounding factors can only affect the heterogeneity of the data distribution. |
| Results | | | | |
| Participants | 13* | (a) Report numbers of individuals at each stage of study—eg numbers potentially eligible, examined for eligibility, confirmed eligible, included in the study, completing follow-up, and analysed | 8 | From January 2016 to April 2018, 1361 Chinese underage inpatients received IMP, and 188 patients were included in this study as required. The screening process for patients was shown in Fig 1. Besides, the general conditions of 188 patients were shown in Table 1. |
|  |  | (b) Give reasons for non-participation at each stage | 8 | From January 2016 to April 2018, 1361 Chinese underage inpatients received IMP, and 188 patients were included in this study as required. The screening process for patients was shown in Fig 1. Besides, the general conditions of 188 patients were shown in Table 1. |
|  |  | (c) Consider use of a flow diagram | 8 | From January 2016 to April 2018, 1361 Chinese underage inpatients received IMP, and 188 patients were included in this study as required. The screening process for patients was shown in Fig 1. Besides, the general conditions of 188 patients were shown in Table 1. |
| Descriptive data | 14* | (a) Give characteristics of study participants (eg demographic, clinical, social) and information on exposures and potential confounders | 8-10 | Line 158-169 |
|  |  | (b) Indicate number of participants with missing data for each variable of interest | 8 | Fig 1 |
|  |  | (c) *Cohort study*—Summarise follow-up time (eg, average and total amount) | N/A |  |
| Outcome data | 15* | *Cohort study*—Report numbers of outcome events or summary measures over time | N/A |  |
|  |  | *Case-control study—*Report numbers in each exposure category, or summary measures of exposure | N/A |  |
|  |  | *Cross-sectional study—*Report numbers of outcome events or summary measures | *10-12* | *Line 172-181* |
| Main results | 16 | (*a*) Give unadjusted estimates and, if applicable, confounder-adjusted estimates and their precision (eg, 95% confidence interval). Make clear which confounders were adjusted for and why they were included | N/A |  |
|  |  | (*b*) Report category boundaries when continuous variables were categorized | 4-6 | The reference intervals in Xiangya Hospital, which are based on the reference intervals of adults [15], are ALT, male (9-50 U/L), female (7-40 U/L); AST, male (15-40 U/L), female (13-35 U/L); total bilirubin (TBil), 1.7-17.1 μmol/L; direct bilirubin (DBil), 0-6.8 μmol/L; alkaline phosphatase (ALP), 45-125 U/L.  Definition of abnormal liver function: at least one of ALT, AST, ALP, TBil and DBil was above the upper limit (ULN) of the reference value. Abnormal liver function was graded by classification criteria in NCI.CTC v4.03 [17]. The determination of abnormal liver function level of every patient was subject to the highest one in 4 indexes.  The Naranjo adverse reaction scale [18] was used to grade the included patients, and the possibility of variances of liver function laboratory indexes induced by IMP was evaluated according to the score. The possibility was assigned to four categories from the total score as follows: Definite, ≥9; Probable, 5 to 8; Possible, 1 to 4; Doubtful, ≤0. |
|  |  | (*c*) If relevant, consider translating estimates of relative risk into absolute risk for a meaningful time period | N/A |  |

Continued on next page

| Other analyses | 17 | Report other analyses done—eg analyses of subgroups and interactions, and sensitivity analyses | 12 | Line 184-191 |
| --- | --- | --- | --- | --- |
| Discussion | | | | |
| Key results | 18 | Summarise key results with reference to study objectives | 8-14 | Line 158-214 |
| Limitations | 19 | Discuss limitations of the study, taking into account sources of potential bias or imprecision. Discuss both direction and magnitude of any potential bias | 20-21 | Line 354-362 |
| Interpretation | 20 | Give a cautious overall interpretation of results considering objectives, limitations, multiplicity of analyses, results from similar studies, and other relevant evidence | 21 | Line 365-369 |
| Generalisability | 21 | Discuss the generalisability (external validity) of the study results | 19-20 | Line 332-352 |
| Other information | |  | | |
| Funding | 22 | Give the source of funding and the role of the funders for the present study and, if applicable, for the original study on which the present article is based | N/A, but in submission system | **Financial Disclosure: The authors received no specific funding for this work.** |

*Give information separately for cases and controls in case-control studies and, if applicable, for exposed and unexposed groups in cohort and cross-sectional studies.

**Note:** An Explanation and Elaboration article discusses each checklist item and gives methodological background and published examples of transparent reporting. The STROBE checklist is best used in conjunction with this article (freely available on the Web sites of PLoS Medicine at http://www.plosmedicine.org/, Annals of Internal Medicine at http://www.annals.org/, and Epidemiology at http://www.epidem.com/). Information on the STROBE Initiative is available at www.strobe-statement.org.
